# Pyoderma Gangrenosum: The Impact of Treatment Non-adherence on Disease Progression

**DOI:** 10.7759/cureus.51490

**Published:** 2024-01-01

**Authors:** Carmen Iliescu, Liliana Popa, Mara Mihai, Marius N Popescu, Cristina Beiu

**Affiliations:** 1 Dermatology, Elias Emergency University Hospital, Carol Davila University of Medicine and Pharmacy, Bucharest, ROU; 2 Oncological Dermatology, Elias Emergency University Hospital, Carol Davila University of Medicine and Pharmacy, Bucharest, ROU; 3 Physical Medicine and Rehabilitation, Elias Emergency University Hospital, Carol Davila University of Medicine and Pharmacy, Bucharest, ROU

**Keywords:** inflammatory skin disorders, chronic wounds, neutrophilic dermatoses, non-healing ulcer, pyoderma gangrenosum

## Abstract

Pyoderma gangrenosum (PG) is a rare, ulcerative, rapidly progressing, destructive, inflammatory cutaneous disease that is both diagnostically and therapeutically challenging. Due to the lack of standardized diagnostic criteria or conclusive guidelines for patient management, clinicians often find themselves without reliable tools for the daily management of PG patients. Additionally, the lack of strict therapeutic compliance in patients with this diagnosis might contribute to a catastrophic evolution of the condition. We report a case of ulcerative PG that is illustrative of the inherent challenges posed by patients frequently changing healthcare providers and treatment regimens, displaying inconsistency and non-adherence. Such behaviors can lead to the loss of disease control, particularly in the context of extensive or rapidly progressing PG, ultimately culminating in the development of mutilating forms of this disease.

## Introduction

Pyoderma gangrenosum (PG) is a rare inflammatory disorder falling under the wide spectrum of neutrophilic dermatoses. It has an estimated incidence of 3-10 patients per million people per year, with females being slightly more predisposed than men to develop PG [1].

PG frequently co-occurs with various systemic conditions, most commonly inflammatory bowel diseases, hematologic malignancies, monoclonal gammopathy of undetermined significance, and rheumatologic disorders (such as psoriatic arthritis and rheumatoid arthritis) [2]. In most cases, the clinical presentation is consistent with the classical appearance of an inflammatory papule, pustule, or nodule that progresses to an extremely painful erosion or ulcer with a violaceous border and necrotic base, predominantly on the lower legs [3]. Given the absence of specific clinical, laboratory, or histopathologic findings, the diagnostic process often presents a challenge. This challenge can lead to diagnostic ambiguity, treatment delays, and even patient resistance to accepting the diagnosis or the appropriate treatment.

We present a complex clinical scenario of PG in a 35-year-old Caucasian female, marked by challenging diagnostic and therapeutic decisions. The case illustrates the severe consequences of non-adherence to treatment, resulting in rapid and extensive disease progression, including toe amputation and exposure of vital tissues within the ulcerative lesions.

## Case presentation

We report the case of a 35-year-old Caucasian female who presented in our dermatology department in November 2022 with a one-year history of two non-healing ulcers on the anterior and lateral aspects of the left shin and a two-month history of an enlarging ulcer on the exterior aspect of the right lower leg. The lesions initially presented as very tender papules and pustules that rapidly expanded and progressed into ulcers over two months, without any history of preceding trauma.

The patient also suffered from a gastric ulcer and duodenitis, diagnosed last year. She underwent two colonoscopies that revealed no signs of inflammatory bowel disease. No other significant medical issues were reported. The patient had not been using any medications, and her lifestyle habits were unremarkable, except for a noteworthy habit of heavy smoking.

Before presenting in our department, the patient was examined in various centers, which suspected the diagnosis of PG, but three consecutive biopsies were inconclusive for a definitive diagnosis, showing mainly nonspecific vasculitic findings. However, several institutions prescribed systemic treatment with multiple conventional immunosuppressive drugs, such as azathioprine, methotrexate, and cyclophosphamide, along with corticosteroids, therapies that the patient followed only for several weeks before discontinuing at her own discretion.

**Figure 1:**
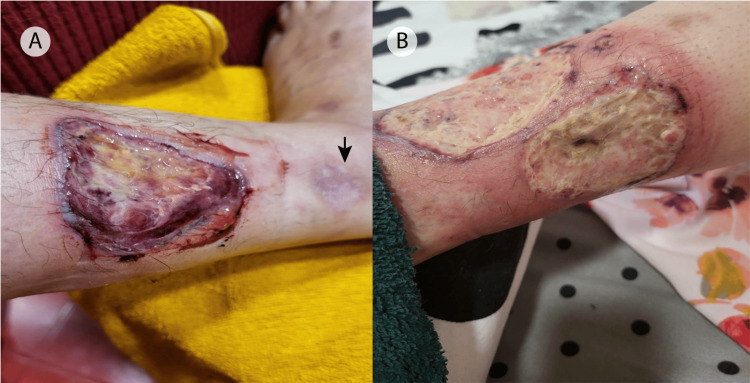
Clinical findings A single purulent ulcerative lesion with asymmetrical, undermined violaceous borders is located on the exterior aspect of the right lower leg (A). Two similar ulcers are located on the anterior and lateral aspects of the left shin, with the tendency to coalesce into one larger lesion (B). An atrophic, cribriform scar (black arrow) is noticeable at the site of a previously healed lesion (A).

During her first hospitalization in our clinic in November 2022, the physical examination revealed multiple ulcers on both the right and left shins, extremely tender with a necrotic base, purulent discharge, and a violaceous undermined border. An atrophic, cribriform scar was also noticed at the site of a previously healed ulcer (Figures 1A,B). The clinical aspect was suggestive of PG. Except for a body mass index of 15.24 kg/m², the rest of the examination was unremarkable.

An extensive serologic evaluation was completed, which included a complete blood count, peripheral blood smear, serum immunofixation electrophoresis, erythrocyte sedimentation rate, C-reactive protein, a comprehensive metabolic panel including liver and renal function tests, and an autoimmune panel including antinuclear antibody, rheumatoid factor, antineutrophil cytoplasmic antibodies, antiphospholipid antibodies, and C3 and C4 complement. Laboratory investigations were normal, except for the presence of an inflammatory reaction and mild iron deficiency anemia. Arterial and venous Doppler ultrasound results were within normal limits. Bacterial cultures of samples from the ulcerative lesion on the right leg were positive for *Staphylococcus aureus*.

**Figure 2:**
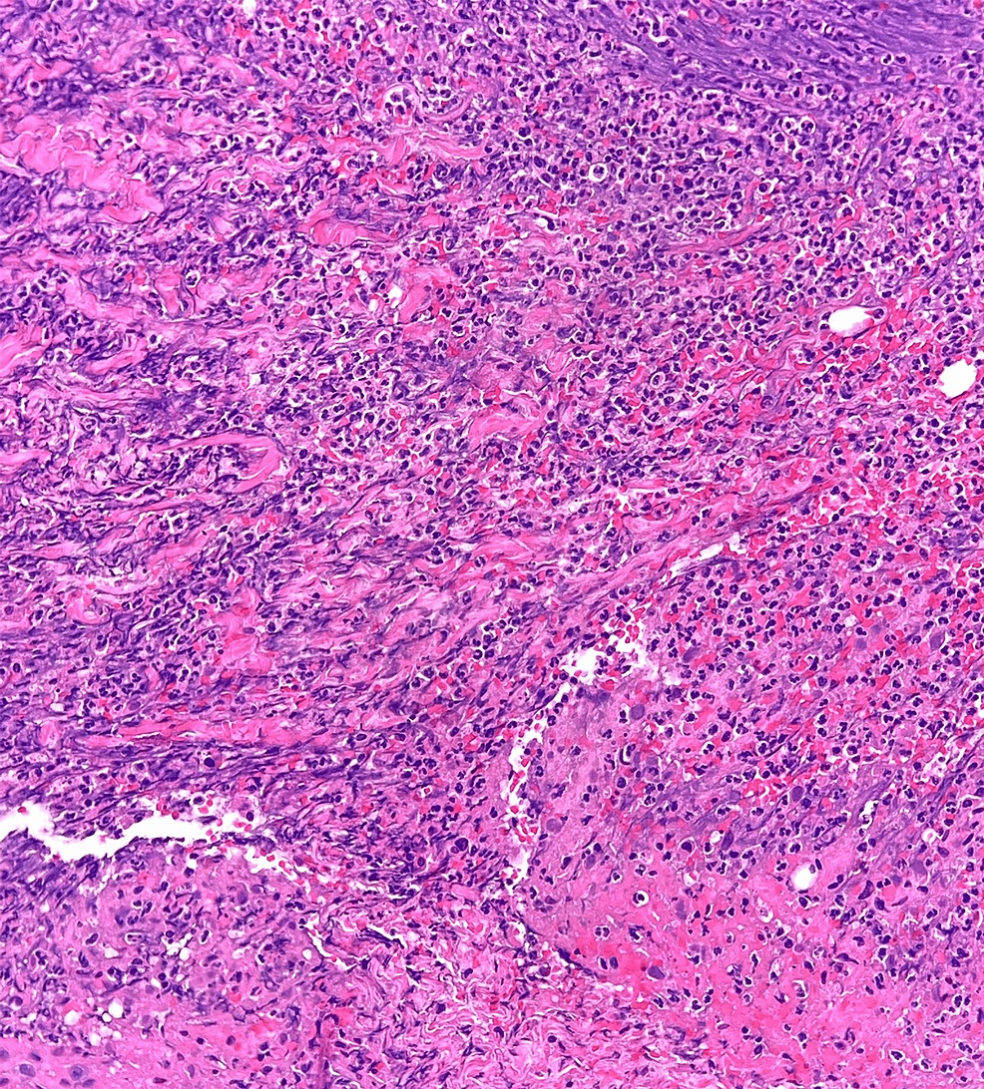
Histopathology displaying dense neutrophilic dermal infiltrate.

An elliptical incisional biopsy of the ulcer's inflammatory edge, extending deep into the subcutaneous fat, was performed, including histological stains and cell cultures. It showed a diffuse sterile neutrophilic dermal infiltrate, interstitial hemorrhage, edema, and fibrosis, all consistent with PG (Figure 2).

During her hospital stay, the patient was started on dexamethasone 8 mg per day for rapid control of the ulcer's extent and systemic dapsone 100 mg/day as a non-immunosuppressive adjunctive steroid-sparing agent. Systemic cyclosporine at an initial dose of 4 mg/kg was highly recommended, but the patient refused any forms of supplementary immunosuppressive therapy. Local treatment with antiseptic solutions and modern wound dressings (alginate and polyurethane foam) was also introduced into the therapy regimen. She was discharged from the hospital with a treatment plan consisting of systemic glucocorticoids (prednisone 50 mg/day, with gradual dose reduction), dapsone 100 mg/day, and twice-daily local applications of tacrolimus 0.1% ointment.

**Figure 3:**
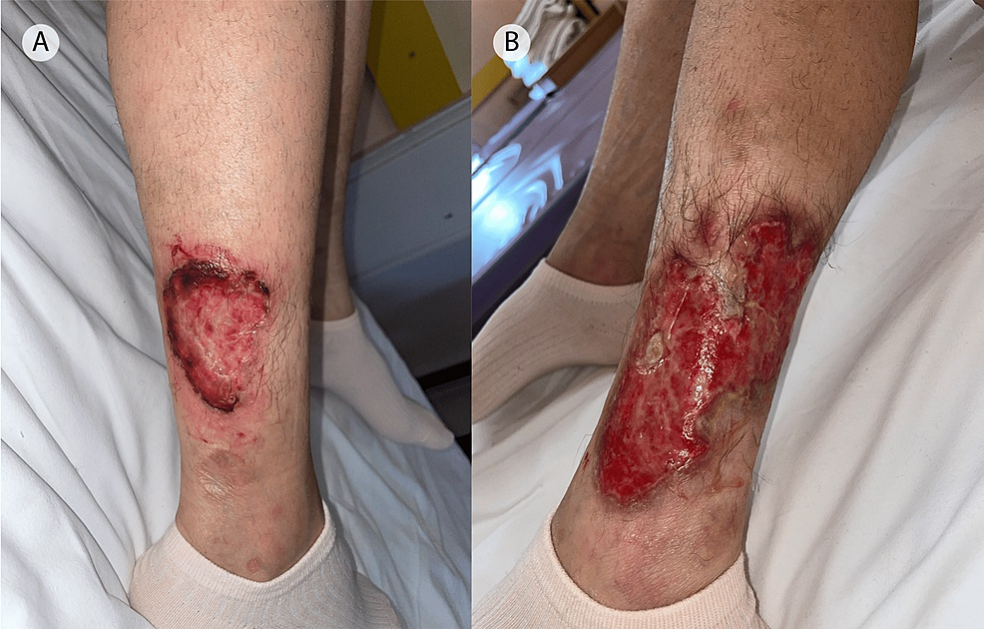
Clinical findings showing a marked improvement in the ulcers of the patient after one month of treatment. The initial purulent and necrotic base was gone, and a tendency toward healing could be noticed (A, B).

At the one-month follow-up visit, we observed a marked improvement in purulent discharge and a significant decrease in the size of the ulcers, with clinically visible signs of healing (Figures 3A, B).

**Figure 4:**
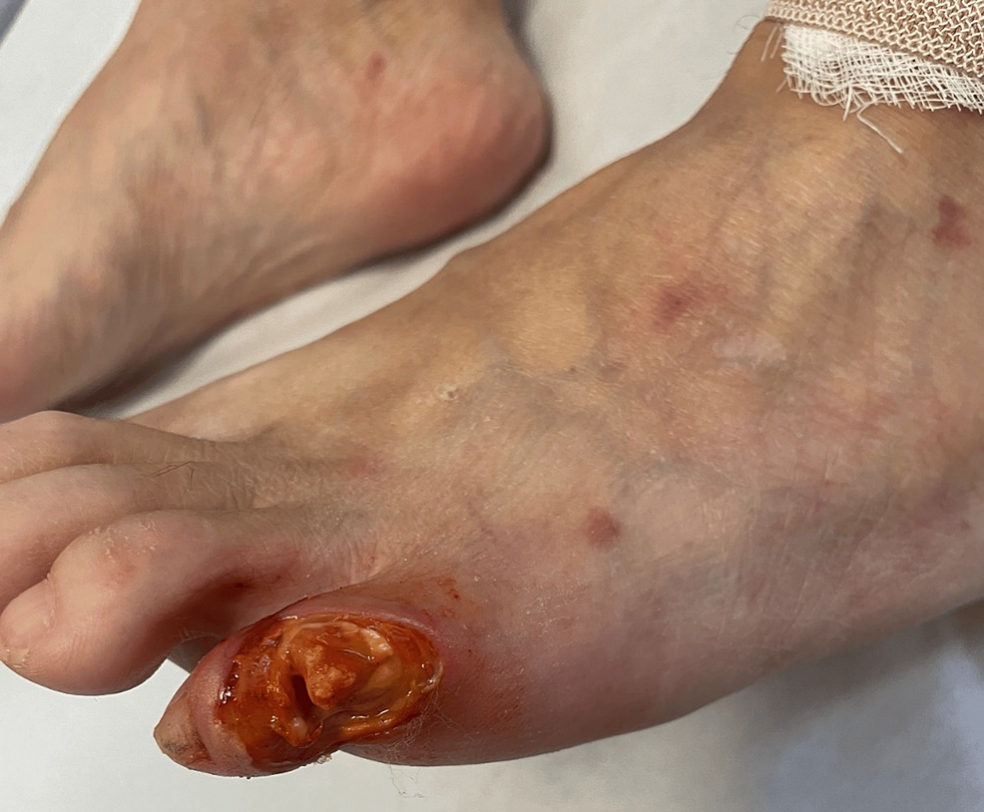
Deep ulcerative lesion with a violaceous border. Exposure of the distal phalanx of the left fifth toe.

In January 2023, the patient presented again in our clinic with a new ulcerative lesion located at the site of a previously excised painful callus on the fifth toe of the left foot (Figure 4). She was referred to the plastic surgery department where, unfortunately, the distal phalanx could not be saved due to exposure of vital tissue in the ulceration bed, and amputation was performed. On her own choice, the patient decided to stop taking dapsone and she still refused any form of immunosuppressive therapy. At that point, she was also lost to follow-up.

**Figure 5:**
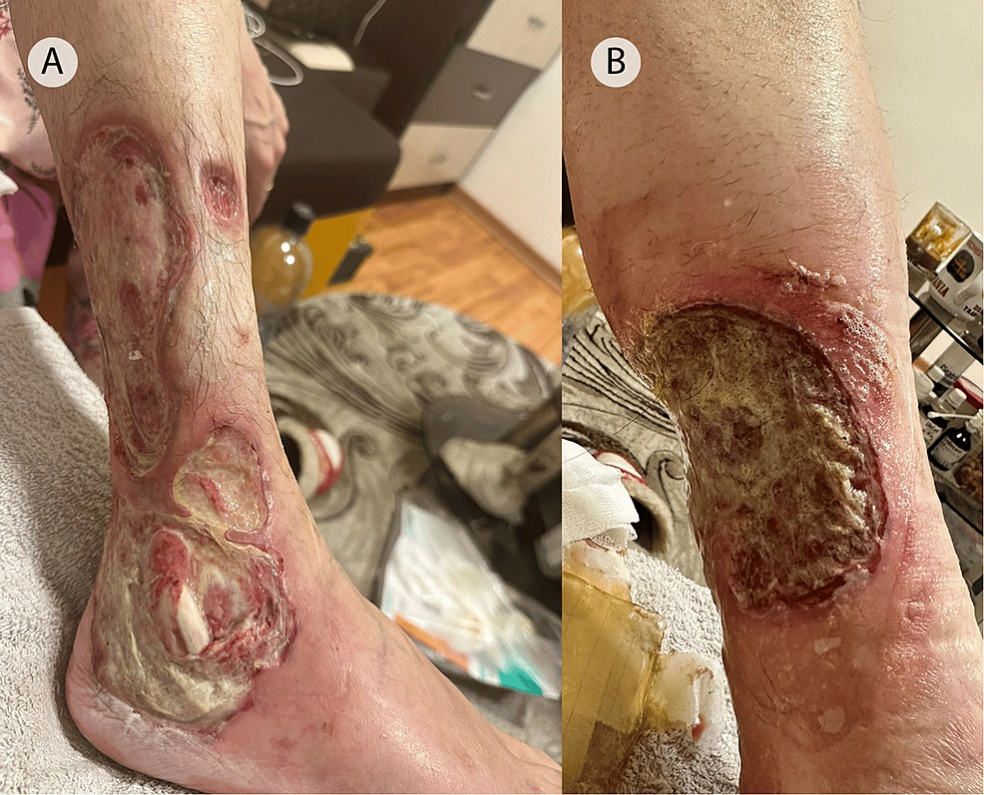
Multiple ulcerative lesions with irregular enlargement resulting in a serpiginous shape. The exposed peroneal tendon is visible in the purulent base of the ulcer (A). Pyoderma gangrenosum lesion with extensive necrosis of the skin extending to the gastrocnemius muscles (B).

She returned to the outpatient unit of our department in April 2023. Unfortunately, in the absence of treatment, the lesions of PG rapidly progressed. New ulcers developed, and the old ones expanded in size and depth. The process was so profound that the peroneal tendon was exposed (Figures 5A, B). The patient was referred for surgical intervention and was again lost to follow-up.

## Discussion

We report a rapidly progressing, destructive case of PG and emphasize the critical importance of strict therapeutic compliance and continuous, careful monitoring in patients with this condition to reduce the risk of catastrophic evolution.

This case report focuses on a 35-year-old Caucasian woman, aligning with the typical demographic profile seen in patients with PG. According to the existing literature, PG most commonly emerges in young to middle-aged adults and tends to have a higher incidence among women [1].

The patient's medical history revealed a history of gastric ulcer and duodenitis, and despite the patient's two prior colonoscopies, a definitive diagnosis of inflammatory bowel disease (IBD) was not established. It is essential to note that more than 50% of patients with PG have an associated inflammatory bowel disease, and in some cases, PG may precede the formal diagnosis of an associated disorder. As such, ongoing follow-up is crucial to monitor the patient for the possible development of IBD [2].

To begin with, establishing the diagnosis of PG is very challenging due to its variable clinical presentation and nonspecific laboratory or histopathologic findings, with the diagnosis being one of exclusion. In our case, the patient history, the clinical characteristics, and the presence of diffuse neutrophilic dermal infiltrate in the biopsy specimen outlined the diagnosis, thus fulfilling one major criterion and five minor criteria for ulcerative PG according to the Delphi Consensus of International Experts (Table 1) [4].

**Table 1:** Fulfilled criteria for pyoderma gangrenosum.

Type of criteria	Features
Major criterion	Histopathology of ulcer edge showing a diffuse neutrophilic dermal infiltrate
Minor criteria	The absence of infection
	Medical history of inflammatory arthritis
	Papulopustular lesions that rapidly degenerated into ulcers
	Extremely painful ulcerations, with violaceous and undermined edges
	Numerous ulcerations affecting the lower legs
	Cribriform scar at the site of the previously healed ulcer

In the process of diagnosing this case, we encountered several diagnostic challenges and considered a range of differential diagnoses. These included the possibility of arterial occlusive and venous diseases, vasculitis, infections, and malignancies. To narrow down these possibilities, an extensive panel of antibodies was meticulously examined, and the results were found to be within normal limits. This comprehensive evaluation helped us rule out the presence of an autoimmune vasculitic process.

We also considered the potential involvement of arterial and venous diseases. To explore this further, we conducted Doppler ultrasound assessments, which, notably, yielded normal results. While these investigations were crucial in ruling out arterial and venous diseases, it is important to highlight that obtaining a biopsy is a common practice to assess the presence of alternative causes of ulcerations, as recommended in the existing literature [4]. In our specific case, we employed special histopathologic staining techniques to meticulously investigate the possibility of an infectious process. This step was instrumental in thoroughly evaluating the presence of any infectious etiology, ultimately contributing to the diagnostic process.

Even after establishing the correct diagnosis of PG, there are no firm patient management guidelines because there is a lack of information on interventions for PG. The majority of treatment strategies focus on minimizing inflammation while providing suitable wound care and pain management [5]. Examples of regularly used drugs include topical corticosteroids, topical calcineurin inhibitors, intralesional corticosteroids, systemic corticosteroids, conventional immunosuppressants, and biologics. While topical therapy is sufficient for the local form of the disease, systemic therapies, such as systemic corticosteroids and immunosuppressants, are suggested for recalcitrant local or extended disease [5].

For PG that does not respond well enough to first-line therapy, a wide range of additional systemic immunomodulatory medications can be used as an alternate or supplementary treatment. Examples include dapsone, minocycline, and conventional immunosuppressants [6]. Infliximab has shown efficacy for PG in a randomized trial, and given that it is also useful for Crohn's disease, it is preferred for usage in people with both conditions [7].

The most effective regimens still comprise a combination of medications that are customized to the patient's condition and socioeconomic status, with the vital requirement of maintaining strict adherence to the treatment regimen. Studies suggest that, with appropriate treatment, over 50% of individuals diagnosed with PG may achieve full wound recovery within a year, and the majority experience remission with longer-term follow-up [8].

However, as seen in our case, a lack of therapy adherence can lead to a devastating evolution. Ulcerative lesions can progress in size and depth within the subcutaneous fat and subsequently lead to exposure of underlying tendons, ligaments, or even bones, predisposing the patient to a potentially lethal infection [5,9].

In advanced cases, the necessity for surgical procedures arises, potentially initiating a challenging cycle, given the well-established controversy surrounding the role of surgery in PG due to its potential to induce pathergy. To mitigate this risk, it is advisable to restrict surgical procedures to phases of effective disease management, while also providing patients with concurrent systemic therapy [10], emphasizing the continued importance of treatment adherence.

In our patient's case, it is crucial to acknowledge her perspective and her reasons for discontinuing systemic treatments. The patient expressed feelings of insecurity and frustration, primarily stemming from the uncertainty surrounding the management of PG. As the medical literature indicates, PG presents a diagnostic and therapeutic challenge, with no standardized treatment protocols available [5]. The concern was exacerbated by the absence of high-quality efficacy studies for the various therapies prescribed to her.

The patient's decision-making process was also notably influenced by her desire to observe tangible signs of improvement within a relatively short timeframe. She opted to discontinue treatments if she did not perceive noticeable improvements within weeks of initiating them. This preference for rapid results is understandable and reflects the patient's yearning for relief from the distressing symptoms of PG. However, it is important to emphasize that the nature of PG treatment often requires patience, as ulcer healing typically takes weeks to months [6].

Another significant factor in the patient's decision-making process was her concern about the potential risks associated with long-term treatment, particularly corticosteroids and systemic immunosuppressive therapies. This concern is valid, as these therapies are indeed linked to a risk of serious adverse effects [3].

To address this concern and enhance patient understanding, healthcare providers must engage in open and transparent communication with patients diagnosed with PG. Patients should be clearly informed that while some treatments may yield initial signs of improvement within days, the journey to ulcer healing often necessitates several weeks to months. Additionally, it should be emphasized that systemic therapies, including corticosteroids and immunosuppressive agents, should be gradually tapered and stopped over several months, rather than being abruptly discontinued.

## Conclusions

In conclusion, PG is a highly unpredictable and potentially devastating disorder that demands careful attention and management. This case report highlights the rarity and severity of PG and the significant impact of treatment adherence. It showcases the optimal response achieved with systemic corticosteroids and dapsone, in stark contrast to the disfiguring progression when treatment adherence was compromised. Additionally, this case underscores the multifaceted challenges in PG management, including the patient's decision-making influenced by a desire for rapid improvement and concerns about treatment risks. With the absence of standardized treatment protocols and limited high-quality efficacy data, patient education and transparent communication become pivotal. Balancing treatment efficacy and safety is essential, emphasizing the crucial role of effective patient-provider collaboration in navigating the complexities of PG and achieving positive outcomes.
